# Overlap between birth trauma and mistreatment: a qualitative analysis exploring American clinician perspectives on patient birth experiences

**DOI:** 10.1186/s12978-023-01604-0

**Published:** 2023-04-21

**Authors:** Cynthia Salter, Kristina Wint, Jessica Burke, Judy C. Chang, Patricia Documet, Elizabeth Kaselitz, Dara Mendez

**Affiliations:** 1 Department of Behavioral and Community Health Sciences, University of Pittsburgh School of Public Health, 6135 Public Health Building, 6th Floor Public Health,130 DeSoto St, Pittsburgh, PA 15216 USA; 2 Association of Maternal and Child Health Programs, 1825 K St NW, Washington, DC 20006 USA; 3 Department of Obstetrics, Gynecology & Reproductive Sciences, Internal Medicine, and the Clinical and Translational Science Institute, University of Pittsburgh School of Medicine, 300 Halket St., Pittsburgh, PA 15213 USA; 4 Department of Epidemiology, University of Pittsburgh School of Public Health, 5130 Public Health, 130 DeSoto St, Pittsburgh, PA 15216 USA

**Keywords:** Birth, Birth trauma, Psychological birth trauma, Mistreatment, Disrespect, Respectful maternity care, Human rights in birth

## Abstract

**Introduction:**

Research exploring the mistreatment of birthing people in the United States is emerging rapidly within the context of increasingly poor maternal health outcomes that include unacceptable racial disparities. Previous research has explored overlap between psychological birth trauma and mistreatment using patient descriptions of birth experiences, but no previous studies have explored these issues from the perspectives of clinicians. The aim of this study was to explore whether maternity care providers’ descriptions of patient birth trauma overlap with categories of mistreatment from a globally accepted typology.

**Methods:**

Content analysis was performed on a qualitative data set of 28 semi-structured interviews about patient birth trauma, completed in 2018–2019 with U.S. maternity care clinicians, including obstetricians, family physicians, midwives and labor/delivery nurses. The interviews were part of a larger study exploring maternity clinician perspectives and experiences of patient birth trauma. For this analysis Krippendorff’s method of categoric distinction was used, with categories from a globally recognized typology of maternity patient mistreatment.

**Results:**

Clinicians’ descriptions of their experiences with patient birth trauma mapped onto all seven mistreatment categories, although no interview questions specifically asked about mistreatment. In more than 30 hours of interviews, transcribed to more than 800 pages, the word mistreatment appears only once, suggesting that some healthcare providers may use the phrase “birth trauma” as a euphemism to describe mistreatment. Eighteen of 28 interviews included at least one description that fit into a mistreatment category. “Failure to meet professional standards of care” was the category with the most mapped clinician statements, followed by “Stigma and discrimination” and “Poor rapport between women and providers.”

**Conclusions:**

This study contributes new insight into maternity clinicians’ conceptualization of patient trauma and how their descriptions of birth trauma overlap with mistreatment. Clinicians implicitly connected mistreatment with some patient experiences of birth trauma, even when they were not specifically asked about mistreatment. Findings point to a need for further research into mistreatment, including routinized “everyday care” that may include mistreatment, particularly for marginalized and historically excluded birthing people. Future research also must explore the potential role of mistreatment in poor and inequitable U.S. birth outcomes.

## Plain language summary

Many people giving birth in the United States experience poor health outcomes, and there is a wide racial disparity, with people of color more likely to experience poor outcomes. In recent research, birthing people reported that they were mistreated during their labor and delivery, including being shouted at, scolded, or threatened. Mistreatment accounts were more frequent among women of color. Previous research has looked at patient reports about their birth experiences to explore whether their descriptions of psychological trauma include overlap with mistreatment, but no other studies have looked at descriptions of birth trauma from the perspectives of medical clinicians. The objective of this study was to explore whether maternity care providers’ descriptions of patient birth trauma overlap with categories of mistreatment from a globally accepted list. This study analyzed the content of 28 semi-structured interviews about patient birth trauma, completed in 2018–2019 with obstetricians, family physicians, midwives and labor/delivery nurses. In the interviews, participant descriptions of patient birth trauma fit into all seven mistreatment categories. Participant descriptions included examples of patients receiving medical procedures or treatments without first giving consent, nurses avoiding the rooms of patients who do not speak English, and other forms of mistreatment. Participants were not asked specifically about mistreatment, but they described birth trauma by giving examples of mistreatment, which suggests that some healthcare providers may use the phrase “birth trauma” when talking about “mistreatment.” This study shows a need for further research into mistreatment, including routine “everyday care” that may include mistreatment.

## Introduction

Research exploring the mistreatment of people giving birth in the United States is emerging rapidly within the context of increasingly poor maternal health outcomes that include unacceptable racial disparities. In a 2019 online survey of 2,700 mothers, one in six U.S. mothers (17%) reported experiencing some form of mistreatment during delivery, including loss of autonomy, being shouted at, scolded or threatened, or being ignored [1]. Mistreatment was more frequent among women of color—32.8% for indigenous women, 25% for Hispanic women, and 22.5% for Black women. Among women of color with low socio-economic status (SES), 27.2% reported mistreatment, compared to 18.7% of low SES white women [1]. A recent online survey with 748 women from Australia, Europe and North America reporting psychological birth trauma found “the majority (66.7%) described care provider actions and interactions as the traumatic element in their experience”[2], pointing to overlap between patient experiences of psychological birth trauma, which is more widely researched in high-income countries, and mistreatment, for which a broad literature exists for low- and middle-income countries [3].

In the United States, care of birthing people and their newborns is the most common reason for hospitalization, accounting for the highest percentage of hospital costs billed to both private and public insurance [4]. Most of the 3.6 million U.S. births each year take place in hospitals [5, 6], yet pregnancy-related outcomes are shockingly poor. The U.S. maternal mortality ratio (MMR) is 23.8 pregnancy-related deaths per 100,000 live births [7], the highest among high-income countries, compared to France (8.7), Canada (8.6), the United Kingdom (6.5), Germany (3.2), and New Zealand, (1.7) [8]. Among U.S. non-Hispanic Black women the MMR is 2.9 times higher, at 55.3 deaths, compared with 19.1 deaths among non-Hispanic White women [7]. The COVID-19 pandemic further exacerbated poor U.S. birth outcomes and widened disparities [9].

Birthing people also experience serious and potentially long-lasting health effects. Severe maternal morbidity (SMM), including cardiovascular and hypertensive conditions, has been steadily increasing in the U.S. and compromises health for thousands of post-partum people [10]. SMM includes post-traumatic stress symptoms (PTSS), reported by one-third of birthing people[11, 12], with smaller percentages (1.7 to 9%) meeting psychiatric criteria for post-traumatic stress disorder (PTSD) [13]. Postnatal trauma symptoms have severe effects on the health of birthing people and their families in the short and long term [14–17], and postnatal trauma symptoms interfere with infant attachment and breastfeeding [15, 16, 18, 19], and may result in other long-term poor outcomes for infants and children as well as birthing people [20].

The World Health Organization endorses a human rights-based approach for reducing morbidity and mortality among birthing people, advocating care “that maintains their dignity, privacy and confidentiality, ensures freedom from harm and mistreatment, and enables informed choice and continuous support during labour and childbirth” [21]. In 2015, Bohren et al. used thematic synthesis to distill maternity patient descriptions from 65 studies in 34 countries into “an evidence-based classification system of how women are mistreated during childbirth in health facilities” [3]. The resulting typology includes seven broad types of mistreatment [3] (Table 1). In 2018 a U.S. trauma researcher used this typology to analyze patient birth trauma descriptions and found that patient narratives could be categorized into six of the typology’s seven forms ofpatient mistreatment [14]. That study and others [2, 3, 14, 22, 23] previously have explored overlap between birth trauma and mistreatment using patient descriptions of birth experiences. Most recently, a Dutch study used the same mistreatment typology to complete a qualitative content analysis of 438 social media birth narratives and paired that content analysis with a separate inductive coding analysis [24]. That 2020 study reported that ineffective communication, loss of autonomy and lack of informed consent/confidentiality were the most often described forms of mistreatment. As in that study, the content analysis reported here is part of a larger study that included inductive thematic analysis. However, because this is the first study where maternity care clinicians describe patient mistreatment, we elected to focus on the Krippendorff’s content analysis for this report. We are unaware of any studies that have explored maternity care mistreatment from the perspectives of clinicians.

**Table 1:** Typology of mistreatment [3]

First-order themes	Second-order themes	Third-order themes
Physical abuse	Use of force Physical restraint	Women beaten, slapped, kicked, or pinched during delivery Women physically restrained to the bed or gagged during delivery
Sexual abuse	Sexual abuse	Sexual abuse or rape
Verbal abuse	Harsh language Threats and blaming Harsh language	Harsh or rude language Judgmental or accusatory comments Threats of withholding treatment or poor outcomes Blaming for poor outcomes Harsh or rude language
Stigma and discrimination	Discrimination based on sociodemographic characteristics Discrimination based on medical conditions	Discrimination based on ethnicity/race/religion Discrimination based on age Discrimination based on socioeconomic status Discrimination based on HIV status
Failure to meet professional standards of care	Lack of informed consent and confidentiality Physical examinations and procedures Neglect and abandonment	Lack of informed consent Breeches of confidentiality Painful vaginal exams Refusal to provide pain relief Performance of unconsented surgical operations Neglect, abandonment or long delays Skilled attendant absent at time of delivery
Poor rapport between women and providers	Ineffective communication Lack of supportive care Lack of autonomy	Poor communication Dismissal of women’s concerns Language and interpretation issues Poor staff attitudes Lack of supportive care from health workers Denial or lack of birth companions Women treated as passive participants during childbirth Denial of food, fluids or mobility Lack of respect for women’s preferred birth positions Denial of safe traditional practices Objectification of women Detainment in facilities
Health system conditions and constraints	Lack of resources Lack of policies Facility culture	Physical conditions of facilities Staffing constraints Staffing shortages Supply constraints Lack of privacy Lack of redress Bribery and extortion Unclear fee structures Unreasonable requests of women by health workers

## Methods

### Study aim and setting

This study explored potential overlap between maternity care clinicians’ descriptions of patient birth trauma and an accepted typology of mistreatment analyzed, using qualitative inquiry [25]. The data set included verbatim transcripts of 28 qualitative interviews completed with maternity care clinicians who provide birth service in a midwestern U.S. city of 303,000, where the metropolitan population racial make-up is 65% non-Hispanic white, 23% Black or African American, and 11% Asian, other or two or more races [26]. The [BLINDED UNIVERSITY IRB] approved this research (PRO 17020090).

### Sampling and recruitment

Sampling was purposive and drew from three maternity care settings: a university-affiliated maternity care hospital, a smaller suburban hospital and a free-standing birth center. Recruitment involved two stages: (1) Five maternity clinicians serving as community recruiting partners publicized the study using email and flyers with the heading “Are you a maternity healthcare provider interested in birth trauma?” (2) We used snowball sampling by asking participants to introduce the study to other clinicians who might be interested in participating. Obstetricians, family physicians, midwives and labor/delivery nurses providing delivery care were eligible to participate.

### Data collection

Semi-structured interviews were conducted between November 2018 and June 2019, using an interview guide that was developed based on a comprehensive literature review and formative discussions with the five maternity clinician community partners. The guide included open-ended questions and was field-tested and then revised to incorporate feedback from the five community partner maternity clinicians.

Participants gave verbal consent and completed in-person interviews in locations of their choosing between November 2018 and June 2019. The first author, a trained and experienced qualitative interviewer, conducted the interviews, which explored clinicians’ perceptions and experiences of “birth trauma” among their patients, using open-ended questions (See Table 2). Interviews were audio-recorded and transcribed verbatim by a transcription service. Participants completed a demographic questionnaire and received literature about secondary trauma among clinicians, with a local support phone number, and a $10 gift card.

**Table 2:** Sample interview questions

• When you hear the word trauma in relation to birth, what do you think of?
• Tell me a little bit about your own experiences as a [physician, midwife, nurse,] with birth trauma
• Let’s talk a little bit about the delivery room and the practice setting at your hospital [birth center]. What kinds of things do you think might impact whether or not the patient perceives the birth as traumatic? What kinds of things might impact whether the provider finds the birth traumatic?

### Data analysis

Interview transcripts, participant demographic data, and transcript coding were managed in NVivo 12 [27]. The interviewer compared transcriptions with audio recordings for accuracy and corrected when necessary. After completing a separate initial thematic analysis on clinician perceptions of birth trauma [25], (the results of which will be summarized in a separate, forthcoming report), a content analysis was completed based on Krippendorff’s content analysis of categoric distinction [28]. This analysis used as the unit of analysis the study participants’ narrative statements about their experiences with patient birth trauma. Categories for content analysis were taken directly from the 2015 “Typology of mistreatment of women during childbirth” [3] (Table 1). The first author completed initial categorization of participant statements, while secondary authors reviewed the categorization of participant statements to interrogate and confirm findings. Results reported here are confined to the research findings from the Krippendorff’s content analysis that was completed using the 2015 Bohren et al. typology of mistreatment.

## Results

Twenty-eight maternity healthcare professionals completed interviews, including labor and delivery room nurses (n = 14), certified nurse midwives (n = 8), and physicians (n = 6). Interview length averaged 54 min (range = 34–74 min). Participant median age was 36 (range = 24–64), and average time working in their current maternity care capacity/setting was 6.9 years (median 4 years, range = 1–28 years). See Table 3 for participant demographics.

**Table 3:** Demographic characteristics of participants (n = 28)

Variable	N	%
Profession		
Labor and Delivery Nurse	14	50
Certified Nurse Midwife	8	28.5
Physician	6	21.4
Gender		
Female	27	96.4
Male	1	3.6
Race/Ethnicity		
White/European	24	85.7
Black/African American	1	3.6
Asian/Pacific Islander	1	3.6
Multi-Race	1	3.6
Hispanic/Latinx	1	3.6
Married/Partnered	17	60.7
Single	11	39.3
Previously Given Birth		
Overall	17/27*	62.9%
Nurses	6/14	42.8%
Midwives	7/8	87.5
Physician	4/5*	80.0

*1 male participant excluded from calculations about giving birth

During the interviews about patient birth trauma, many maternity care clinicians described patient experiences that meet global classifications of mistreatment, although study participants did not specifically label the experiences as mistreatment. In more than 30 hours of interviews, transcribed to more than 800 pages, the word mistreatment appears only once. Eighteen of 28 clinicians (64%) described at least one experience of patient birth trauma that mapped onto a mistreatment category.

Clinician narratives of their experiences with patient birth trauma mapped onto all seven categories in the mistreatment typology. In our initial categorization, we did not map any clinician statements to the category of “sexual assault.” However, upon review, it was pointed out that at least one clinician’s description of a painful vaginal examination meets the Federal Bureau of Investigation’s criteria for sexual abuse. Specifically, “penetration, no matter how slight, of the vagina or anus with any body part or object, or oral penetration by a sex organ of another person, without the consent of the victim” is considered sexual assault [29]. Therefore we, revisited the transcripts and included this category here. Individual category descriptions and examples of clinician statements are presented below.

### Failure to meet professional standards of care

This mistreatment category, which includes lack of consent, painful vaginal examinations, and refusal to provide pain relief, along with other care short-comings, had the most clinician narratives mapped to it, including multiple statements from 7 different clinicians (25% of total sample) (2 midwives, 3 nurses and 2 physicians). Lack of consent was the most frequently described patient experience in this category. Participants described situations with colleagues who did not introduce themselves to patients, and described seeing other maternity clinicians perform cervical exams, cut episiotomies, rupture membranes, and perform vacuum-assisted deliveries without obtaining consent, without explaining the procedure, and, sometimes without patients being aware of what had taken place.

A midwife said:

*“… watching some of my physician colleagues who I respect and who do a really great job—cut women’s bodies without their consent—I have a hard time with that”* (#10, midwife).

A physician described this situation:

*Some people, though, rush through that, and they like, start wheeling the patient back to the operating room, and are like, “We’re going to do a C-section right now” and they’re assuming they’re getting verbal consent from the patient. And all of a sudden, the patient is like, “What’s going on...”* (#24, obstetrician).

A nurse described a patient whose bag of waters was being broken:

*She kept telling him to stop and he didn’t stop. And they [the family] refused to let him back in the room.* (#14, nurse).

### Stigma and discrimination

This category includes discrimination based on sociodemographic characteristics, and the study’s second-highest number of clinician statements mapped onto this category of mistreatment. Statements were from 6 different providers (21.4%) (2 midwives, 2 nurses and 2 physicians). One physician and one nurse described subtle differences in the way patients are described during transitions of care:

*When the patients have an attitude about something it comes off differently if they’re one race versus another race … a Black patient who has an attitude is like, “Oh, God [*strained voice*] she’s so horrible, don’t go in that room” versus ... we have [a White patient] right now who wants us to deliver her at thirty-four weeks… But … the descriptive word for her is “needy” … [*laughs*] versus like “attitude”* (#27, obstetrician).

One nurse described how some staff avoided patient rooms of women of color or patients who do not speak English:

*... I think if there was a fly on the wall for report, even at the beginning of shifts, you can just kind of tell which patients the nurses care about more and which ones are just a “pain in the ass” or like, “not worth the time”* (#3, nurse).

Two midwives described situations where providers underestimated serious medical conditions of their patients of color when they sought emergency care. One said:

*The two patients that we’ve had, that have had really bad, concerning, postpartum depression who didn’t receive adequate treatment were both Black ... They were sent home from the ER with postpartum preeclampsia that they could’ve died from!* (#29, midwife).

### Poor rapport between patients and providers

This category includes ineffective communication, including dismissal of birthing people’s concerns as well as poor maternity care staff attitudes. Statements from 6 different providers (21.4%) (1 midwife, 2 nurses and 3 physicians) mapped to this category, which includes ineffective communication, lack of supportive care and lack of autonomy. One obstetrician said:

*So, in the middle of her C-section she started having terrible pain and anesthesia was giving her more meds ... I was like “call your attending ... she’s in excruciating pain.” ... and the attending came in and then he [the resident] was like “Oh, she’s just having … pain with motion.” And, I was like, “No, … she shouldn’t be moving her legs” … And it turned out that her epidural catheter had actually fallen out of her back … she actually had essentially an un-anaesthetized C-section”* (#27, obstetrician).

Language or translation issues fall within this category, and a nurse said that using the translation phone with non-English speaking patients was seen as a “*time-suck*,” leading some nurses to avoid interacting with non-English-speaking patients:

*Those [patients] require more work. And it’s not that it’s difficult work. But when your staffing ratios are terrible, it’s harder...it’s a time-suck. And you have other patients* (#16, nurse).

### Health system conditions and constraints

This category includes the facility culture as well as limitations such as policies or staffing constraints and appeared in statements from 5 clinicians (17.8%), including 2 midwives, 2 nurses and 1 physician. Participants at one hospital described how some providers mocked patients who had birth plans or whose birth preferences were outside what staff considered to be mainstream. For example, one heard coworkers say: *Yeah, she has a birth plan so we’re taking bets on how soon it’s going to be before she’s sectioned* (#6, nurse).

Respondents said hospital policies had differential effects on some groups of patients:

*The visitor policy, I don't think, is always culturally acceptable to … Black families that tend to want … more of their family members there … people are being asked to leave just because they're over the number of people that are allowed* (#13, midwife).

An obstetrician practicing at a Federally Qualified Health Center said some of her patients of color or Latinx ethnicity are singled out by hospital drug screening policies, which mandate screening if patients miss multiple prenatal visits.

*... But some of it ... has to do with people’s socioeconomic status too, because, for example, here they will do a urine drug screen on anyone who has had less than five [prenatal] visits … I have several patients who’ve had less than five visits ... They’ll have visits scheduled; they won’t come. And I don’t think they need drug screens. They just need transportation* (#27, obstetrician).

### Verbal abuse

This category includes harsh language, judgmental or accusatory comments, threats of withholding treatment, threats of poor outcomes, and blaming patients for poor outcomes, and statements from 4 clinicians (14.3%) mapped to it (2 midwives, 1 nurse and 1 physician). A physician described this patient’s experience:

*The anesthesiologist yelled at her cause she couldn’t sit still and told her if she couldn’t stop moving, she wouldn’t get an epidural, and walked out of the room* (#27, obstetrician).

One nurse described her own behavior threatening a woman of color with poor infant outcomes:

*I mean, sometimes you’ve got to be a little rough … We’re not really supposed to, but sometimes that’s what you got to tell them … “If you do this, if you leave against our medical advice, your baby’s going to die”* (#19, nurse).

### Physical abuse

This category includes use of force, and one nurse’s narrative mapped onto this category:

*One of the nurses that was a charge nurse … pushed me out of the way and grabbed the woman’s leg, and was in her face and screaming, “You’re not pushing ...” She [the patient] was doing everything she could, and I couldn’t help but think, “what is this woman [*the charge nurse*] thinking? … if a charge nurse … was meaner to her then maybe she could’ve done it right?”* (#4, nurse).

### Sexual abuse

This category includes sexual abuse and rape, and as described earlier, in our initial categorization we did not map any clinician statements to this category. Upon review the following statement was included in this category.

A nurse described this situation:

*I’ve seen ... patients getting exams, and they’re screaming … because it’s so uncomfortable. At the time, like you don’t think any better cause … we need to check their cervix ... when they tell us, “Stop. Stop. Stop. It hurts. It hurts.” And the provider keeps going just because they need to get that exam, and they’re not thinking ... they’re doing anything wrong...* (#19, nurse).

## Discussion

Clinicians implicitly connected mistreatment with some patient experiences of birth trauma, even when they were not specifically asked about mistreatment. This study’s findings of overlap between clinician descriptions of birth trauma and the globally accepted typology of maternity mistreatment suggest that some healthcare providers may use the phrase “birth trauma” as a euphemism to describe mistreatment. Although clinicians were asked about birth trauma, in many cases they described patient mistreatment that they had experienced. These research findings point to a need for further exploration into providers’ implicit conceptualizations of patient mistreatment and birth trauma.

This is not the first study in which participants have drawn connections between birth trauma and mistreatment; however, previous studies elicited patient perspectives, and this is the first study in which examples of mistreatment are described by U.S. maternity clinicians. Their narratives closely mirror patient descriptions in prior birth trauma research, where patients were asked about their experiences of birth trauma and described inattentive or even hostile treatment by healthcare personnel [18, 23, 30, 31], lack of consent [23, 30, 32], inadequate patient information [30, 33], and dissatisfaction with maternity care as contributors to their birth trauma [34]. Additionally, participants in our study describe situations where patients were treated differently because of race, ethnicity or lack of English-language skills, similar to descriptions in recently published literature focusing on disrespect and abuse, mistreatment and racism [1, 2, 23, 35–40], which have been shown to contribute to poor maternity outcomes [41]. As some clinicians, policymakers and even general readers might tend to discount patient accounts of mistreatment, our results, with descriptions of mistreatment coming from clinicians themselves, lend weight to mistreatment research.

Research into mistreatment in maternity care settings has been focused in low- and middle-income countries, while in the U.S. and other high-income countries wider research has explored birth trauma, using a psychological trauma framework, and categorizing patient narratives of mistreatment as “subjective distress”[42] and “patient-provider interaction hotspots”[43]. This framework has been useful for exploring treatment needs, including the need for adequate patient support at birth to mitigate traumatic responses and the higher risk of trauma for patients with prior mental health conditions. However, as noted earlier, one in six U.S. mothers (17%) reported some form of mistreatment during delivery in a recent survey, and for women of color, that rate was even higher [1]. Clearly, patient mistreatment in U.S. maternity settings requires more explicit exploration and focused intervention.

The 2020 Dutch study of social media content that applied the same typology of mistreatment to 438 respondent birth stories found multiple examples of statements by birthing people that mapped to all seven categories of mistreatment, further demonstrating that mistreatment during maternity care experiences is not confined to low- and middle-income countries [24]. More recently, a 2022 Australian study explicitly asked birthing people, through an online survey, “Do you think you experienced obstetric violence (dehumanized treatment or abuse by health professionals toward the body or reproductive process of women)?” and reported that 11.6% of 8,546 survey participants responded “yes” or “maybe” to the question [44].

Although all recent research about mistreatment is drawn from patient descriptions, the findings from our study demonstrate that clinicians are willing discuss patient mistreatment, at least as it is related to possible patient outcomes, such as psychological birth trauma. Given that our study did not recruit participants based on a question about mistreatment, but rather about birth trauma, further investigation is required into how to best engage clinicians in productive discussion about this important topic.

Mistreatment in maternity care previously has been labeled a “blind spot” in global efforts to improve maternal health outcomes and ensure person-centered care [14, 45, 46]. A 2014 Lancet series called disrespect and abuse within maternity care systems “a crisis of quality and accountability suggesting failure and injustice at many levels, including injustice to clinicians providing treatment [45]. One United Kingdom study exploring maternity patient-provider interactions noted that routine maternity care can cause harm and urged greater exploration into the impact of “unconscious and unintentional” mistreatment [47].

The Institute of Medicine defines “patient-centered care” as “providing care that is respectful of, and responsive to, individual patient preferences, needs and values, and ensuring that patient values guide all clinical decisions” [48]. However, the U.S. structure of maternity care may present particular challenges for maintaining patient-centered care. Described by Liese et al. (2020) as “rigid technocratic protocols”[23], maternity care in some settings has become routinized to the point that those involved can forget it involves intimate, and often prolonged, patient-provider interaction, as well as a team of multiple clinicians that changes over time. The idea that routine maternity care can cause harm is, understandably, foreign to many clinicians trained in and accustomed to U.S. maternity care practices. Bringing widespread attention to the systemic mistreatment of birthing people in even the most basic of care practices is an important first step in addressing this issue.

Emerging information about patient mistreatment is not unique to maternity care, and exploration continues into potential contributing factors, including clinician burnout, compassion fatigue, and scarcity of resources or time [49–51]. Mistreatment in maternity care settings, however, has its own complex origins, and it must be explored within the larger context of gender discrimination, intersectionality [52–54], violence against women, structural violence [52, 54], and the racist and exploitative history of obstetric care [37, 55, 56].

Interventions to address obstetric mistreatment must consider not only the role of clinicians but also the systems in which they operate [51], to avoid subsequent mistreatment of healthcare providers [54], who are themselves often subject to high amounts of stress, fatigue, and mistreatment by healthcare systems [23]. It is important to note the potential personal/professional risks that clinicians can face when calling out colleagues’ inappropriate behavior in the hierarchical maternity care system. However, naming and confronting obstetric mistreatment and violence does not necessitate judgment of clinicians, but rather, facilitates accountability and change [57].

This study contributes to a broader public health understanding of mistreatment of birthing people in the U.S. healthcare setting. Choosing to analyze maternity healthcare professionals’ narratives of birth trauma was a deliberate attempt to describe clinician conceptualizations of birth trauma and to explore overlap with mistreatment. The insight from this study into potentially routinized mistreatment of birthing people is timely and crucial given the current poor U.S. maternal outcomes and persistent stark racial inequities.

## Limitations

These results are based on a single qualitative study of 28 clinicians recruited from one geographical region and interviewed once. Completing more interviews with a more diverse sampling of clinicians and including follow-up interviews may have elicited different results. Participants had a stated interest in patient birth trauma, and thus may differ from general maternity clinician populations. Patient factors such as age, race and ethnicity may be further associated with patient experiences of disrespect and mistreatment, and this study did not specifically explore differences along these variables. This study also did not include triage staff and anesthesiology staff, two groups whose importance in setting the tenor of maternity care surfaced in many interviews. Future research should include clinicians from these specialties and explicitly explore how mistreatment varies across racial, ethnic, and socio-economic groups.

## Conclusion

Clinicians implicitly connected mistreatment with some patient experiences of birth trauma, even when they were not specifically asked about mistreatment. Their descriptions point to the need for further research into mistreatment in maternity care, including routinized “everyday care” that may include mistreatment, particularly for marginalized and historically excluded birthing people. Future research must explore the prevalence of mistreatment and its potential role in poor and inequitable U.S. birth outcomes. Initiatives to improve the mistreatment of birthing people must be multifaceted and must consider the structural mistreatment of healthcare workers, as well as longstanding, routinized obstetric practices, racism, classism and other biases in care provision.

## Data Availability

The datasets generated and analyzed during this study are not publicly available to maintain participants’ anonymity and confidentiality, but are available from the corresponding author on reasonable request.

## Appendix

### Semi-structured interview guide

Maternity care providers’ perspectives and experiences of birth trauma-[Author REDACTED]

#### Semi-structured interview field guide

[Begin **here** after *Verbal Consent* is completed, questions are answered, and demographic questionnaire is completed].

My goal is to explore providers’ perspectives and experiences with birth trauma. I am interested in what you think, so please feel free to be honest and to share your point of view. The purpose of this interview is to gather your thoughts, perspectives, and opinions. You may also want to provide examples from your own practice or share your own experiences or even those of colleagues. In order to complete all the questions, there may be times when I redirect the conversation back to the bigger questions, but I want to remind you that there are no right or wrong answers to these questions. Feel free to stop me at any time if you have questions. Also, remember that you do not have to answer any questions that you don’t want to answer, and you can end the interview at any time if you no longer want to participate. Can we proceed with the questions?

[response] Thank you

[Begin recording]

Today is [date] and I am beginning interview number [ID# from completed demographic questionnaire] with a participant who has agreed to be interviewed and to have our conversation audio-recorded.

#### Broad perceptions and conceptualization of birth trauma

1. **When you hear the word trauma in relation to birth, what do you think of?** [Prompts:]
  - How would you describe this situation or how would other [physicians, midwives, nurses] in your field describe these kinds of situations?
  - Is this something that everyone experiences? Is it something that you and your colleagues talk about it? How about in case meetings or in-service trainings?
  - How are experiences like this documented?
  - Do you think a patient could perceive the birth as traumatic, even when from a medical point of view it might seem to be a pretty typical experience or have minimal or no adverse outcomes?
    i. Can you share your thoughts about those situations?

#### Participants’ experiences or experiences of colleagues

2) **Tell me a little bit about your own experiences as a [physician, midwife, nurse, resident] with birth trauma.** [Prompts:]
  - How do you think the patient responded to that situation? Tell me about your own response?
  - What in your training prepared you for this kind of situation?
  - What do you think could have helped the situation to turn out differently?
  - An experience with traumatic birth might stay with a [physician, nurse, midwife, resident] after the immediate situation. Can you talk a little about that?
    i. What do you think might impact the provider’s response (positive and negative)? (or magnify the chances of it staying with you?)
    ii. What about traumatic birth and burnout? Can a traumatic birth experience cause a [physician, nurse, midwife, resident] to consider leaving maternity care?
  - What role do you think lack of support resources might play in burnout after traumatic birth? What about time pressures to get back to work after traumatic births?
  - What are some of the challenges in talking to others about these situations?
  - What about feeling the need to maintain a professional role in traumatic situations—how does that impact a provider’s reaction?
    i. How might fear of litigation come into play?
    ii. What do you think could mitigate these issues?
  - What about a patient who perceived her birth as traumatic, but in which the event appears to have been a normal birth with no poor outcomes, yet the mother reports trauma? Have you experienced anything like this?

#### Influence of practice setting, care environment, health system, society

3) **Let’s talk a little bit about the delivery room and the larger health care environment at your hospital [birth center]. What kinds of things do you think might impact whether or not the mother perceives the birth as traumatic? What kinds of things might impact whether the provider find the birth traumatic?** Prompts:
  - Let’s talk a bit about the immediate birth setting. What do you think could impact the potential for trauma there for either the patient or the provider?
  - What about the roles of different staff in the room? Tell me more about your role? What about the others involved during delivery?
  - How do you think the patient’s interactions with others during the birth might impact her perceptions of the birth as traumatic?
  - What role do you think the partner’s support plays in these kinds of situations?
  - Let’s talk a little bit about communication. How does that come into play in a potentially traumatic birth situation?
  - What factors impact communication with the mother?
  - What do you find about your delivery setting that helps you to do your job and to work best with your patients at birth? What gets in the way?
  - Let’s talk a little bit about the care environment and the practice setting, beyond the immediate birth room. What other care environment factors would you say impact the patient’s response to birth? For example, I am wondering about staffing levels and hospital policies and time pressures, high census—those sorts of things. What do you think?
  - Are there any things that you wish were handled differently or that you think could be reworked to support you and your patients better?

#### Moving forward, how can we address this issue for patients & providers?

4) **Thinking back on the topics we’ve discussed, the broad idea of birth trauma, the potential for women to experience trauma even when it seems nothing goes wrong during birth, the experiences that you and other colleagues have had over the course of your career and the potential for burn-out, what do you think needs to be done?** Prompts:
  - What do you think is the most urgent part of this issue to address first? Why do you say that?
  - What sorts of resources do you think you and your peers need? What about any specialized training? Is any of that available? Tell me more about that.
  - And what sorts of resources do you think patients need? What resources are available for your patients? How do you let patients and providers know about these resources?
  - How might the practice type or practice make-up impact what happens after the birth in terms of making resources available to the both the provider and patient?
  - What parts of this situation do you think providers can address? How?
  - Who else needs to be involved to address this problem?

#### Closing

5) **My final question: what else do you think it would be helpful for me to know about this topic that I haven’t asked about yet?** [Prompt:]
  - Is there anything else you would like to add?

Thank you for your willingness to talk with me today and for sharing your insights about the research topic. We have covered a lot of information and I am grateful that you have shared your experiences with me.

Thank you
